# Nurses' Work Interruptions and Responses: Multitasking During Medication Tasks and Differences by Nursing Delivery System

**DOI:** 10.1155/jonm/2439568

**Published:** 2025-09-05

**Authors:** Kyung Jin Hong, Eunhee Lee

**Affiliations:** ^1^College of Nursing, Kangwon National University, Chuncheon 24341, Republic of Korea; ^2^College of Nursing, Sungshin Women's University, Seoul 02844, Republic of Korea

## Abstract

**Aim:** This study aimed to report not only the frequency and source of work interruptions but also nurses' responses to them, focusing on multitasking during medication-related work. It also examined the differences in work interruption according to the nursing care delivery system.

**Background:** Work interruptions increase nurses' workload complexities and errors, as they compromise nurses' attention.

**Design:** The time and motion cross-sectional observational study method was used.

**Method:** Data were collected between September 4 and 15, 2023, from six nurses in a functional nursing care delivery unit and five nurses working eight-hour shifts in an integrated nursing care unit at a general hospital in South Korea. The Work Observation Method by Activity Timing program was used to record the tasks performed by nurses.

**Results:** The number of interruptions, including multitasking per hour, was 11.21 and 9.27 for the functional nursing care delivery unit and the integrated nursing care unit, respectively. The most interrupted locations were the nurse station (41.6%) and patient rooms (35.4%) in each unit, respectively. Common sources of interruptions were other nurses and patients in both units, followed by family or caregivers in the functional nursing delivery unit and equipment malfunction in the integrated nursing care unit. Nurses responded to work interruptions during medication administration, multitasking 2.79% and 3.02% of the time they administered medication in the functional nursing care delivery unit and integrated nursing care unit, respectively. The most common task in multitasking with medication involved communicating with other professionals.

**Conclusions:** Interruptions and multitasking disrupt medication-related tasks in healthcare environments and require strategies to mitigate frequent work interruptions.

**Implications for Nursing Management:** Efforts aimed at mitigating interruptions that disrupt patient safety, including preventing medication errors and developing interventions based on differences in the nursing delivery system, are needed.

## 1. Introduction

Nurses interdependently perform nursing activities with various personnel, and interruptions during tasks are unavoidable. An interruption is defined as a temporary break in human activities [1], and nurses' experiences range from 0.4 to 13.9 interruptions per hour [2]. Previous research has indicated that interruptions occur most frequently in pediatric units, and other healthcare providers are the most common source of interruptions for nurses working in intensive care units and general wards [2, 3]. However, a study conducted in an emergency department found that nurses' work was frequently interrupted by interactions with patients and their families [4]. Nurses have reported embracing interruptions as an inherent component of clinical care because of their coordinator role and feel as if they are constantly “working in a minefield” [5] and there work is a “big chain of interruptions” [6]. Interruptions occurred most often during times surrounding nursing reports and less often during times when fewer nursing tasks were being performed, such as between 12 o'clock and 4 o'clock on either day or night shift [6], and experienced nurses who can manage and control them are less likely to be upset by interruptions because of their ability to prioritize tasks quickly and perform some tasks automatically [7].

In some cases, interruptions are necessary, as they may relate to providing an overview of patient-related situations, work procedures, knowledge sharing, competence development, and collaboration, which can help nurses work more efficiently [7]. In addition, interruptions are needed to provide help for urgent requests [8] and build rapport with patients to make them sufficiently comfortable to talk to nurses about things that they may not have discussed with a physician [6]. Moreover, some interruptions may contribute to increased safety and comfort of patients and improve nurses' accuracy by providing the recipient with requested information or helping improve patient care [9, 10]. These types of interruptions should be promoted to organize various healthcare personnel related to patient safety.

However, previous research has primarily focused on the negative effects of interruptions, as they could compromise the attention of workers and increase nurses' workloads [2]. Several studies have established that interruptions lead to a constant rearrangement of priorities, which may induce feelings of ineffectiveness and being under the pressure of time; they delay work routines, disrupt reflective processes, and break the continuity of nursing care [10, 11]. Interruptions have also been associated with a higher mental workload, which can be significantly more intense with interruptions during documentation and patient care because nurses are required to respond to multiple tasks against the limited capacity of working memory [12, 13]. Because interruptions are inevitable, increasing research has examined the sources of interruptions and nurses' responses to interruptions during certain tasks [14], focusing on multitasking during work.

Interruptions cause multitasking, which implies engaging in multiple tasks within a limited time. Nurses can become distracted and lose focus on the important aspects of their tasks, which may result in errors. In particular, interruptions during medication events, which are typical in healthcare settings [15], are associated with procedural failures and clinical errors. In interrupted medication events, 34% were found to involve at least one procedural failure [16], and for nurses who received 12 interruptions compared to those who received 3 interruptions, the rate ratio of clinical errors and procedural failures committed was 2.0 and 1.2, respectively [17]. In a qualitative study, nurses failed to give medicine to the patients at the correct time when they faced interruptions and would leave the medicine on the nightstand, which could threaten patient safety [18]. The entire medication process includes prescribing, transcribing, dispensing, and administering medication. Approximately 12% of intravenous medication administration processes are interrupted, mostly by other nurses or patients [19]. This study explains the frequency, multitasking time, and tasking with medication process tasks.

The source and frequency of interruptions and multitasking may differ according to the nursing care delivery system, which is defined as a systematic approach to organizing nursing care for clients [20]. South Korea's nursing systems include functional, team, and primary nursing, with the first two systems being the most common, whereas institutions with low nurse staffing levels prefer functional nursing care delivery systems [21]. However, an integrated nursing care service has been established, aimed at improving the nursing unit environment while providing specialized nursing care from nursing staff without the need for a caregiver [22].

Compared to functional nursing care delivery units, integrated nursing care service units do not include caregivers or family residents but have high nurse staffing levels. Therefore, differences in the frequency and patterns of interruptions between the two nursing care delivery systems are likely to emerge. Exploring these differences through this study contributes to laying the foundation for discussing interruption management strategies according to the nursing care delivery system, given the scarcity of studies reporting differences in interruptions during nurses' tasks according to the nursing delivery system.

This study aimed to (1) report the frequency, source, and response to interruptions during the work of nurses; (2) examine nurses' multitasking while on duty, focusing on medication tasks; and (3) investigate the differences in interruption cases according to the nursing delivery system.

## 2. Materials and Methods

### 2.1. Design and Participants

This prospective observational study was conducted at a general hospital in South Korea between September 4 and 15, 2023. A functional nursing care delivery unit and an integrated nursing care unit were selected to report the differences in nursing care work and interruptions during work, according to the nursing care delivery system. Both operated on a three-shift (day–evening–night), eight-hour working system. Tenured nurses with one to 5 years of experience were recruited to observe their nursing care activities and cases of interruption for each shift on weekdays only. To target only nurses who were mainly involved in direct care, part-time, charge, and night-shift nurses, as well as managers, were excluded.

Nurse managers of the selected observation units were asked to post and guide recruitment documents for participation in the study, and observations were conducted with nurses who agreed to participate. The 22-shift nursing work was conducted by 11 nurses, and the total number of observations was approximately 180 h for the two units. The observation time exceeded that of a previous study that reported nurses' responses to interruptions during medication tasks [14].

### 2.2. Data Collection

#### 2.2.1. Definitions

The definitions of the measured aspects of the observations were based on a previous study that focused on nurses' work and interruptions during tasks [14]. Interruption frequency refers to the number of times a break in the continuity of a task occurred, location refers to the site where the nurse was interrupted, and the source of interruption describes the person or object that initiated the interruption of the original task. Responses to interruption-deferred actions were categorized as switching, mandating, postponing, or rejecting. Switching refers to the suspension of the original task to perform the interruption task. Rejecting implies resuming the original task and verbalizing that the interruption task would not be performed. Postponing refers to the continuation of the original task, with an explanation that the interruption task would be performed later, and mandating refers to the delegation of a task to others. Finally, multitasking is defined as continuing the original task while concurrently performing an interruption task.

#### 2.2.2. Observation Method

Cases and sources of interruptions and nurses' responses were observed and recorded using the Work Observation Method by Activity Timing (WOMBAT) software. Three observers with experience as nurses at a general hospital were recruited, and their tablet computers had a WOMBAT program installed. Approximately 1 hour of training was conducted, which included explaining the purpose and method of observation and how to use the WOMBAT program. Practice observation was performed with the help of a 15-min pilot test video produced by the researcher. The video included a situation with seven instances of interruption, such as discussing patient information with other nurses while preparing medication (multitasking).

### 2.3. Measurements

#### 2.3.1. Nursing Care Activities

Nursing care activities were observed and reported based on the “nursing activities work-sampling instrument” [23]. In this study, nursing activities were categorized as follows: seven types of direct care work, three types of indirect care work, unit-related work, transit, others, and social hours. The WOMBAT program was designed to record nurses' tasks and locations whenever their tasks changed, and the time required for the task could be automatically calculated. The application has two additional buttons that enable interruptions and multitasking to be recorded separately during nursing activities. The source and response can also be recorded in cases of interruption.

#### 2.3.2. Interruption Cases

When an interruption occurred, the observers recorded its source and the nurses' responses, which were categorized as switching, mandating, postponing, or rejecting. The sources of interruption were categorized as other nurses, patients, patients' families or caregivers, alarms, equipment malfunctions, and other workers. When a nurse was multitasking, the observers could select additional tasks that were performed simultaneously in the application program, and when each task was finished, the end button was pressed. The multitasking time was automatically recorded.

### 2.4. Data Analysis

Descriptive statistics, such as frequency and percentage, were used to explain the total observation time and proportion of tasks, number of interruptions, locations of interruption, sources of interruption, responses of nurses, and multitasking time. A chi-square analysis was conducted to report the differences in the locations and sources of interruption according to the nursing care delivery system. An alpha of 0.05 (*p* < 0.001) was used to determine statistical significance. Data were analyzed using Microsoft Excel 2023 and the Statistical Package for Social Sciences, SPSS V. 26.

### 2.5. Ethical Considerations

Before data collection commenced, the research proposal was approved by the institutional review board of the first author's institution (IRB no. KWNUIRB-2023-07-004-001). The researchers obtained consent from nurses who expressed their intention to participate in the study after informing them of its purpose and methods and the reward for participation. They were also informed that they could withdraw their consent at any time. The observers were trained to observe from a distance so as not to interfere with nursing work.

## 3. Results

### 3.1. Comparison of the Observed Nurses' Work and Interruptions

Table 1 presents a comparison of the observed nurses' work and interruptions by the nursing care delivery system. Six and five nursing personnel from the functional nursing care delivery unit and integrated nursing care unit were observed over 12 and 10 shifts, respectively. Nurses in both units allocated more than 50% of their time to direct care activities, followed by indirect care activities, professional communication, unit-related work, transit, and other tasks. The proportion of direct care time was slightly higher in the functional care system (54.4%) than that in the integrated nursing care system (51.2%). Medication administration accounted for the largest proportion in both systems, with functional units requiring more time. Tasks related to hygiene, nutrition, and elimination were notably shorter in the integrated unit. Time spent on indirect care activities was comparable between the two systems. However, documentation took significantly longer in the integrated unit, while the functional unit dedicated more time to handovers and verbal reports. Time allocated to professional communication was similar, accounting for 11.0% and 12.1% in the functional and integrated units, respectively.

The average working hours per shift were similar between the two systems. Actual working hours, excluding social activities, were approximately 7.5 h in both systems. Nurses in functional units experienced significantly more interruptions, averaging 11.21 interruptions per shift compared to 9.27 in integrated units. They were interrupted every 5–6 min, with interruptions occurring more frequently in the functional than in the integrated unit.

### 3.2. Locations and Sources of Interruptions and Nurses' Responses to Them

We analyzed the locations of and responses to interruptions (Table 2). A total of 1096 interruptions were recorded in the functional nursing care delivery unit, compared to 766 in the integrated nursing care unit. The distribution of interruptions across different locations revealed significant variation between the two units (*χ*^2^ = 60.72, *p* < 0.001). In the functional nursing care delivery unit, interruptions most frequently occurred at the nursing station (41.6%), followed by the medical preparation area (24.6%) and patient rooms (22.0%). In contrast, the integrated nursing care unit reported the highest proportion of interruptions in patient rooms (35.4%), with fewer interruptions occurring at the nursing station (27.3%). Regarding responses to interruptions, multitasking was the most common response in both units (50.4% and 47.9% in the functional and integrated nursing care units, respectively), followed by switching (35.9% and 35.1%, respectively). The overall pattern of responses to interruptions did not differ significantly between the two units (*χ*^2^ = 8.36, *p*=0.079).

In addition, we identified the sources of interruptions, excluding those related to multitasking (Table 3). A total of 544 interruptions were observed in the functional nursing care delivery unit and 399 in the integrated nursing care unit. The sources differed significantly according to the nursing care delivery system (*χ*^2^ = 60.72, *p* < 0.001). The most common source of interruptions in both units was other nurses, followed by patients. Notably, interruptions by family members or caregivers were significantly higher in the functional unit (15.3%) than in the integrated unit (0.8%). Other sources included alarms, pagers, phones, equipment malfunctions, and other healthcare workers.

### 3.3. Nurse Responses to Interruptions

Nurse responses to interruptions in functional and integrated nursing care units are presented in Table 4. Direct care activities were the most frequently interrupted in both systems, representing 58.6% and 62.4% of interruptions in the functional and integrated units, respectively. Medication administration constituted the highest proportion of interruptions at 30.8% and 35.0%, respectively. Multitasking was the predominant response during medication tasks, comprising 46.0% and 39.8%, respectively. Indirect care activities accounted for 10.6% and 9.9%, respectively. Recording tasks were frequently interrupted, with multitasking being the dominant response (44 out of 46 interruptions in the functional unit and 34 out of 35 interruptions in the integrated unit). Interruptions during professional communication constituted 18.6% and 16.6% of the total interruptions in the functional and integrated units, respectively. Switching was the most common response, accounting for 37.6% and 29.0%, respectively. Unit-related work, transit, and miscellaneous tasks were interrupted less frequently, but multitasking remained a primary response in both units. For example, unit-related work interruptions accounted for 6.4% in functional units and 2.7% in integrated units, with multitasking comprising 8.9% and 4.1%, respectively.

### 3.4. Duration of and Tasks Involved in Multitasking During Medication-Related Work

In this study, many interruptions occurred during direct care activities, with interruptions occurring most frequently during medication-related tasks.  Table 5 presents the time, location, and tasks performed simultaneously during multitasking owing to interruptions during medication-related work. This study revealed that 2.5 h were spent multitasking during medication-related work, accounting for approximately 3% of the total working hours. Most multitasking occurred in the medication preparation area, comprising 65.4% in the functional unit and 44.5% in the integrated unit. Multitasking with medication predominantly included professional communication, representing 82.0% in a functional unit and 74.0% in an integrated unit.

## 4. Discussion

The nurses observed in this study experienced approximately 10 interruptions per hour, which is relatively high, considering the integrative review by Monteiro et al. [2], reporting a range of 0.4–13.9 interruptions per hour. Notably, compared to Cracker et al. [24], who found that nurses were interrupted approximately every 20 min, this study observed interruptions at a much higher frequency, occurring every 5–6 min. Frequent interruptions during nursing activities can be attributed to the complex and dynamic nature of the healthcare environment in which nurses are required to manage multiple responsibilities simultaneously.

The high frequency of interruptions observed in this study can also be explained by the characteristics of general units, where nurses are typically responsible for 8 to 12 patients. Notably, nurse staffing is much lower in functional care units than in integrated care units, which have doubled their nursing staff in existing nursing units [25]. Although this study did not capture actual nurse-to-patient ratios at the time of observation, the lower frequency of interruptions in the integrated care unit may reflect the benefits of better staffing. As the number of assigned patients increases, nurses must manage a greater volume of tasks, including direct patient care, documentation, medication, and coordination with other healthcare professionals, thus increasing the risk of interruptions due to frequent shifts in attention. In addition, patient-related factors, such as acuity, clinical needs, and care complexity, likely vary across units and may independently influence interruption frequency. Similar patterns have been reported in emergency departments, where high patient volumes and a wide range of urgent tasks contribute to frequent work interruptions [4, 12]. Knight et al. [26] emphasized that interruptions result from a dynamic interplay between urgency, task importance, interruption cost, and team efficiency. Hence, the decision to interrupt or not is shaped not only by staffing levels but also by care complexity, patient characteristics, and the broader clinical environment.

This study observed work interruptions across two types of units, revealing differences in work environments, such as the presence of family caregivers [25, 27]. Functional nursing units, characterized by more frequent caregiver interactions, reported significantly more interruptions than integrated nursing care units. Communication with patients, families, and other healthcare professionals has been identified as a major source of interruptions [4, 28, 29], supporting the finding of increased interruptions in functional units in this study. A key structural distinction is that integrated nursing care units typically restrict caregiver access [25], which may partially explain the lower frequency of caregiver-related interruptions in these settings. The presence of family caregivers inherently increases the likelihood of interruptions during nurses' tasks, as supported by multiple studies conducted in pediatric units, where caregiver involvement is often unavoidable. For example, Zhao et al. [30] found that parents of hospitalized children frequently intervened in the care process, which substantially contributes to nursing interruptions. Hence, clinical environments in which caregivers are expected or required to remain at the patient's bedside, such as pediatric wards or general adult units without caregiver restrictions, may structurally predispose nurses to more frequent work interruptions.

More than 80% of the interruptions in this study resulted in multitasking or switching, indicating that the original tasks were likely not completed with sufficient concentration. Such interruptions can prevent nurses from completing their initial tasks, thereby increasing the risk of missed nursing care, particularly when they are unable to return to their original task. Previous studies have consistently shown that interruptions contributing to switching or multitasking are associated with missed nursing care, reduced care quality, and compromised patient safety [31–33]. The risks are particularly heightened when interruptions occur during tasks that significantly impact patient safety, such as administering or prescribing medications [16, 32, 33]. In this study, over 20% of interruptions occurred in the medication preparation area, and more than 30% occurred during medication administration. Interruptions in these critical areas can lead to cognitive overload, causing nurses to lose focus on essential tasks, which increases the risk of error. Additionally, frequent interruptions can contribute to mental fatigue and reduced situational awareness, further compromising patient safety [12]. Therefore, minimizing unnecessary interruptions and enhancing workflow efficiency are essential for improving patient safety and overall quality of nursing care.

In this study, interruptions occurred most frequently during the medication administration process, followed by communication with patients and professional communication. However, nurses' responses to interruptions differed. They most often responded to work interruptions during medication administration by multitasking; however, they responded to interruptions during communication with patients and other professionals by switching. Although medication administration is a high-priority action in terms of patient safety, nurses were more likely to multitask or delay medication to handle interruptions rather than postpone their response to them, which is consistent with the findings of Reed et al. [14].

This study explored not only frequent interruptions during medication administration but also a high rate of performing both the primary and interrupting tasks simultaneously. This finding suggests that the medication administration process is not only frequently interrupted but also performed under distracted conditions, potentially compromising its safety. Interruptions and distractions during medication administration have been shown to negatively impact patient safety by increasing the risk of medication error and missed care [8, 16, 34].

Consequently, several interventions to reduce interruptions have been implemented, demonstrating positive effects in minimizing work interruptions [35, 36]. In addition to intervention programs, the role of physical environmental modifications in reducing interruptions has recently been explored. Huckles et al. [37] evaluated the impact of separate medication rooms on not only the frequency of interruptions during medication preparation but also self-reported medication error rates. Their findings indicated a significant reduction in interruptions and an increase in uninterrupted preparation time. Given that approximately half of the interruptions during the medication administration process in this study occurred in the medication preparation area, physical separation could be an effective strategy to reduce interruptions and enhance medication safety. Moreover, strategies to diminish interruption by wearing a “do not interrupt” medication vest and providing education on how to deal with interruptions can also be considered [35, 38].

Communication has been identified as a major source of interruptions [4, 28, 29], which is consistent with the findings of this study. Approximately 80% of multitasking cases due to work interruptions during the medical administration process involved both the process and communication. Although communication is frequently cited as a source of interruptions, few studies have examined the purpose or urgency of these communications in detail. In practice, the purpose of communication is challenging to identify in real time, making it difficult to distinguish between essential and nonessential interactions. This study also did not categorize communication-related interruptions by purpose or urgency. According to Teige et al. [39], more than half of all interruptions may be avoidable. This suggests that even communication-related interruptions may have characteristics that distinguish between avoidable and essential disruptions. Therefore, a more detailed classification of communication-related interruptions is needed to minimize adverse effects. Furthermore, communication itself was frequently interrupted in this study, which is consistent with previous findings [40]. Interruptions during communication can lead to communication errors, which may pose significant risks to patient safety [39, 41]. At the organizational level, staff must be taught interruption management that involves judging work priorities in the event of interruptions and stopping interruptions when a nurse is performing crucial work related to patient safety, such as administering medication [42].

This study had some limitations. First, data were collected from a limited number of nursing units, all of which were in acute adult care settings, which may affect the generalizability of the findings to other clinical contexts. Second, patient acuity and various environmental factors, such as staffing levels, workflow, task complexity, and team culture, may have been confounding variables that independently influenced the frequency of work interruptions, regardless of differences in care delivery models. We did not fully control for these variables, limiting our ability to isolate and assess the specific impact of the care delivery model on work interruptions. Third, the observational method may have introduced the Hawthorne effect, potentially underestimating the actual frequency of interruptions. Finally, this study investigated the frequency and types of interruptions without analyzing their severity or direct impact on patient outcomes. Future studies should use validated instruments to classify interruptions based on their potential for harm or relevance to primary nursing tasks to provide a more comprehensive understanding of their impact on care quality and patient safety. Despite these limitations, this study provides valuable insights into the patterns of interruptions according to the nursing care delivery system.

## 5. Conclusions

Interruptions are inevitable during nursing work, and nurses are frequently interrupted and compelled to multitask during patient care, resulting in the original tasks not being completed with sufficient concentration, despite some interruptions being necessary to update urgent information and facilitate cooperation. Other nurses and patients were common sources of interruptions, with multitasking identified as the most common cause of interruptions during work related to medication administration. The locations and sources of interruptions differ based on the nursing care delivery system; therefore, strategies must be developed for the differentiated control of interruptions. Moreover, further studies should work to differentiate between necessary and unnecessary types of interruptions. Some interruptions are necessary to notify nurses of urgent situations or immediately identify patient safety issues so that healthcare workers can make timely corrections.

## 6. Implications for Nursing Management

Strategies to mitigate interruptions and enhance workflow efficiency to improve patient safety and nursing care quality should be implemented. Physical separation of designated medication preparation areas, the use of “do not interrupt” medication vests, and e-learning interventions for managing interruptions can reduce interruptions during medication-related work. Nursing education should include methods to manage interruptions, such as stopping the source of interruptions until completing the current task or asking another staff member to handle any interruptions. Furthermore, interventions should be based on monitoring not only the frequency and sources of interruptions but also the responses of nurses, which vary based on the nursing delivery system. In this study, nurses working in the functional nursing care delivery unit were often interrupted by patients' families or caregivers, whereas in the integrated nursing care unit, nurses were often interrupted by other nurses. Different solutions should be applied based on the source of the interruption. For example, nurses can be taught communication skills to demonstrate assertiveness and not stop their current tasks when interrupted by other nurses and apologize to patients' families and caregivers and explain why they need to finish the current task before addressing their issue.

## Figures and Tables

**Table 1:** Comparison of observed nurses' work and interruptions by the nursing care delivery system.

Variables	Functional nursing care delivery unit	Integrated nursing care unit
Nursing personnel observed	6	5
Total shifts observed	12	10
Day shifts	5	5
Evening shifts	7	5
Observed working time, h:min:sec (%)		
Total	97:46:43	82:39:51
Direct care	53:11:13 (54.4)	42:17:24 (51.2)
Admission/discharge	2:57:20 (3.0)	1:56:55 (2.4)
Patient assessment (e.g., vital signs)	3:49:00 (3.9)	3:52:24 (4.7)
Hygiene/nutrition/elimination	2:25:19 (2.5)	1:02:39 (1.3)
Medication	31:53:36 (32.6)	24:30:36 (29.7)
Procedures	6:48:43 (7.0)	7:21:12 (8.9)
Communication with patients or caregivers	3:30:00 (3.6)	2:13:46 (2.7)
Other direct care	1:47:15 (1.8)	1:19:52 (1.6)
Indirect care	18:38:47 (19.1)	15:44:38 (19.1)
Recording	6:12:52 (6.4)	9:21:47 (11.3)
Handover/verbal report	5:05:04 (5.2)	0:37:54 (0.8)
Other indirect care	7:20:51 (7.5)	5:44:57 (7.0)
Professional communication	10:45:27 (11.0)	10:01:10 (12.1)
Unit-related work	4:32:37 (4.7)	1:12:30 (1.5)
Transit	1:36:31 (1.7)	0:22:38 (0.5)
Others	0:34:47 (0.6)	4:47:26 (5.8)
Social hours	8:27:21 (8.7)	8:14:05 (10.0)
Average working hours per shift	8:08:54	8:15:59
Actual working hours per shift (excluding social hours)	7:26:37	7:26:35
Interruptions, including multitasking		
Total number	1096	766
Number of interruptions per hour	11.21	9.27
Interval of interruptions (mins)	5.35	6.48

**Table 2:** Locations and responses to interruptions across functional and integrated nursing care units.

Variables	Categories	Functional nursing care delivery unit		Integrated nursing care unit		Chi^2^ (*p*)
Interruptions		1096		766		

Locations	Station	456	(41.6)	209	(27.3)	60.72 (< 0.001)
	Medication prep area	270	(24.6)	177	(23.1)	
	Patient room	241	(22.0)	271	(35.4)	
	Hallway	100	(9.1)	74	(9.7)	
	Others	29	(2.6)	35	(4.6)	

Responses	Multitasking	552	(50.4)	367	(47.9)	8.36 (0.079)
	Switching	394	(35.9)	269	(35.1)	
	Rejecting	60	(5.5)	58	(7.6)	
	Postponing	49	(4.5)	50	(6.5)	
	Mandating	41	(3.7)	22	(2.9)	

**Table 3:** Sources of interruptions excluding multitasking.

Variables	Categories	Functional nursing care delivery unit		Integrated nursing care unit		Chi^2^ (*p*)
Interruptions excluding multitasking		544		399		

Source	Nurses	193	(35.5)	184	(46.1)	60.72 (< 0.001)
	Patients	122	(22.4)	102	(25.6)	
	Family or caregiver	83	(15.3)	3	(0.8)	
	Alarm, pager, or phone	72	(13.2)	39	(9.8)	
	Equipment malfunction	50	(9.2)	45	(11.3)	
	Other workers (e.g., physicians)	22	(4.0)	26	(6.5)	
	Missing	2	(0.4)	0	0.0	

**Table 4:** Nurse responses to interruptions in functional and integrated nursing care units.

Original tasks	Functional nursing care delivery unit						Integrated nursing care unit					
	Total	Switching	Mandating	Postponing	Rejecting	Multitasking	Total	Switching	Mandating	Postponing	Rejecting	Multitasking
	1096	394	41	49	60	552	766	269	22	50	58	367
Direct care	642 (58.6)	182 (46.2)	25 (61.0)	37 (75.5)	54 (90.0)	344 (62.3)	478 (62.4)	159 (59.1)	18 (81.8)	34 (68.0)	55 (94.8)	212 (57.8)
Admission/discharge	12 (1.1)	6 (1.5)	0 (0.0)	1 (2.04)	0 (0.0)	5 (0.9)	12 (1.6)	8 (3.0)	0 (0.0)	1 (2.0)	2 (3.4)	1 (0.3)
Patient assessment (e.g., V/S)	22 (2.0)	4 (1.0)	1 (2.4)	0 (0.00)	2 (3.3)	15 (2.7)	31 (4.0)	9 (3.3)	2 (9.1)	1 (2.0)	3 (5.2)	16 (4.4)
Hygiene/nutrition/elimination	22 (2.0)	7 (1.8)	0 (0.0)	3 (6.12)	4 (6.7)	8 (1.5)	19 (2.5)	9 (3.3)	2 (9.1)	6 (12.0)	1 (1.7)	1 (0.3)
Medication	338 (30.8)	34 (8.6)	6 (14.6)	15 (30.61)	29 (48.3)	254 (46.0)	268 (35.0)	60 (22.3)	9 (40.9)	18 (36.0)	35 (60.3)	146 (39.8)
Procedures	73 (6.7)	16 (4.1)	2 (4.9)	5 (10.20)	4 (6.7)	46 (8.3)	71 (9.3)	23 (8.6)	0 (0.0)	5 (10.0)	7 (12.1)	36 (9.8)
Communication with patients or caregivers	124 (11.3)	85 (21.6)	12 (29.3)	10 (20.41)	8 (13.3)	9 (1.6)	59 (7.7)	41 (15.2)	4 (18.2)	3 (6.0)	6 (10.3)	5 (1.4)
Other direct care	51 (4.7)	30 (7.6)	4 (9.8)	3 (6.12)	7 (11.7)	7 (1.3)	18 (2.3)	9 (3.3)	1 (4.5)	0 (0.0)	1 (1.7)	7 (1.9)
Indirect care	116 (10.6)	11 (2.8)	2 (4.9)	2 (4.08)	1 (1.7)	100 (18.1)	76 (9.9)	4 (1.5)	0 (0.0)	3 (6.0)	1 (1.7)	68 (18.5)
Recording	46 (4.2)	1 (0.3)	0 (0.0)	1 (2.04)	0 (0.0)	44 (8.0)	35 (4.6)	0 (0.0)	0 (0.0)	1 (2.0)	0 (0.0)	34 (9.3)
Handover/verbal report	8 (0.7)	0 (0.0)	0 (0.0)	0 (0.00)	0 (0.0)	8 (1.5)	1 (0.1)	0 (0.0)	0 (0.0)	0 (0.0)	1 (1.7)	0 (0.0)
Other indirect care	62 (5.7)	10 (2.5)	2 (4.9)	1 (2.04)	1 (1.7)	48 (8.7)	40 (5.2)	4 (1.5)	0 (0.0)	2 (4.0)	0 (0.0)	34 (9.3)
Professional communication	204 (18.6)	148 (37.6)	2 (4.9)	6 (12.24)	4 (6.7)	44 (8.0)	127 (16.6)	78 (29.0)	3 (13.6)	12 (24.0)	2 (3.4)	32 (8.7)
Unit-related work	70 (6.4)	15 (3.8)	3 (7.3)	3 (6.12)	0 (0.0)	49 (8.9)	21 (2.7)	5 (1.9)	0 (0.0)	1 (2.0)	0 (0.0)	15 (4.1)
Transit	14 (1.3)	4 (1.0)	2 (4.9)	1 (2.04)	0 (0.0)	7 (1.3)	5 (0.7)	4 (1.5)	0 (0.0)	0 (0.0)	0 (0.0)	1 (0.3)
Others	50 (4.6)	34 (8.6)	7 (17.1)	0 (0.00)	1 (1.7)	8 (1.5)	59 (7.7)	19 (7.1)	1 (4.5)	0 (0.0)	0 (0.0)	39 (10.6)

**Table 5:** Duration of and tasks involved in multitasking during medication-related work.

Variables	Categories	Functional nursing care delivery unit	Integrated nursing care unit
Multitasking response during medication-related work		254	146

Multitasking duration, hr:min:sec		2:43:39	2:30:01

% of multitasking duration in working hours		2.79%	3.02%

Locations	Medication prep area	166 (65.4)	65 (44.5)
	Patient room	56 (22.0)	50 (34.2)
	Hallway	26 (10.2)	24 (16.4)
	Station	4 (1.6)	4 (2.7)
	Other	2 (0.8)	3 (2.1)

Tasks involved in multitasking with medication-related work	**Direct care**	**35 (13.8)**	**16 (11.0)**
	Communication with patients/caregivers	28 (11.0)	0 (0.0)
	Admission/discharge	1 (0.4)	0 (0.0)
	Patient assessment (e.g., vital signs)	2 (0.8)	2 (1.4)
	Hygiene/nutrition/elimination	2 (0.8)	0 (0.0)
	Procedures	0 (0.0)	0 (0.0)
	Other direct care	2 (0.8)	14 (9.6)
	**Indirect care**	**219 (86.2)**	**130 (89.0)**
	Professional communication	208 (81.9)	108 (74.0)
	Recording	0 (0.0)	0 (0.0)
	Handover/verbal report	0 (0.0)	0 (0.0)
	Other indirect care	1 (0.4)	0 (0.0)
	Unit-related work	3 (1.2)	0 (0.0)
	Transit	0 (0.0)	0 (0.0)
	Other indirect care	7 (2.8)	22 (15.0)

*Note:* The bold values represent the totals of direct and indirect care.

## Data Availability

The data that support the findings of this study are available from the corresponding author upon reasonable request.
